# Food and nutrient intake and diet quality among older Americans

**DOI:** 10.1017/S1368980021000586

**Published:** 2021-05

**Authors:** Yeon Jin Choi, Eileen M Crimmins, Jung Ki Kim, Jennifer A Ailshire

**Affiliations:** Leonard Davis School of Gerontology, University of Southern California, 3715 McClintock Avenue #218C, Los Angeles, CA 90089, USA

**Keywords:** Healthy eating, Dietary guidelines, Diet Quality, Socio-ecological framework, USA

## Abstract

**Objective:**

A suboptimal diet and nutritional deficiencies can have important influences on health with significant impact among older adults. This study aims to assess the presence of suboptimal dietary intake among older Americans and identify risk and protective factors influencing diet quality.

**Design:**

Cross-sectional secondary analysis.

**Setting:**

USA.

**Participants:**

A nationally representative sample of 5614 community-dwelling older adults over age 54 in the Health and Retirement Study – Health Care and Nutrition Survey.

**Results:**

Overall, only 10·7 % of respondents had a good quality diet (Healthy Eating Index score 81 and above); the majority had diets considered poor or needing improvement. Less than 50 % of respondents met dietary guidelines and nutritional goals for most individual food groups and nutrients. Respondents with low socio-economic status, fewer psychosocial resources and those who had limited access to healthy food outlets were more likely to have a diet of suboptimal quality.

**Conclusions:**

Efforts to remove identified barriers that put older adults at risk for poor nutrition and to provide resources that increase access to healthy food should be made to encourage healthy eating and enhance diet quality.

A suboptimal diet and nutritional deficiency are thought to be important factors in the development of chronic diseases and mortality. Previous studies have reported that low intake of dairy products, fruits and vegetables, whole grains, seafood, nuts and seeds, fibre, Ca and polyunsaturated fat and high intake of red and processed meat, saturated fat, trans fat, added sugar and Na were associated with increased risk of CVD, metabolic syndrome, cancer and osteoporosis^(1–7)^. In addition to associations with single food groups and nutrients, overall diet quality, which reflects the combination of all foods and nutrients consumed, has been associated with increased risk for chronic diseases and cancer mortality^(8–11)^. In 2016, these dietary risks accounted for 11 % of disability-adjusted life-years lost and 529 299 deaths in the USA, with 83·9 % of these deaths caused by CVD^(12)^.

Diet and nutrition may be more problematic for older adults. A reduced appetite due to disease, pain, decreased need for energy and changes in hormones, sense of smell, taste and vision related to ageing may limit their food intake^(13)^. Changes in the digestive system may reduce the absorption of essential nutrients and lead to malnutrition or nutrient deficiency^(13,14)^. In addition, social and economic changes linked to aging may make healthy eating more difficult for older persons. For example, they may experience financial difficulties due to reduced income or increased medical expenditures, which have been identified as risk factors for poor nutrition^(15,16)^. Decreased mobility^(17–19)^, lack of social support^(17,20)^ and social isolation^(17,21)^ due to the loss of close relationships may also make acquiring and preparing food for older adults more difficult, encourage unhealthy eating behaviours (e.g. meal skipping and low intake of core foods) and place them at risk for consuming poor diets and nutritional deficiencies.

Diet is important in older age, but nutritional content may be lacking as a result of biological and social changes that accompany age. However, much existing research has assessed diet and nutrition in younger populations and focused on limited explanations for dietary differences, such as low socio-economic status^(15,16,18)^ and social resources^(20–23)^, limiting our understanding of the particular circumstances relating to dietary intake among older Americans. Therefore, the objectives of this study are to assess dietary intake of core food groups and nutrients and identify risk and protective factors influencing diet quality in a nationally representative sample of older Americans. Findings of this study will deepen our understanding of the diets of older Americans and determining factors associated with diet will inform the development of public health and nutrition policies and interventions that can promote healthy eating.

## Methods

### Sample and data

We used data from the 2013 Health Care and Nutrition Study (HCNS), a sub-study of the Health and Retirement Study (HRS) that asked about health care access, food purchases and food and nutrition consumption. The HRS is a biennial survey of approximately 20 000 people who are a nationally representative sample of Americans over age 50. A random subsample of 12 418 respondents and their spouse/partners were selected to receive the HCNS questionnaire by mail, of whom 7383 age-eligible respondents (ages 54 and over) responded^(24)^. Data from the HRS core survey and the HRS psychosocial and lifestyle survey on sociodemographic, psychosocial and environmental factors were linked to the HCNS; 260 respondents were excluded from the sample because they could not be linked with psychosocial resources data or neighbourhood food environment data (*n* 7123). We then omitted 1509 individuals who had missing information on the following variables: perceived social support (*n* 995), food insecurity (*n* 364), depression (*n* 137), social contacts (*n* 10) and geographic region (*n* 3). After excluding individuals with missing data, the final analytical sample includes 5614 respondents. Respondents with missing data were more likely to be male, Black, Hispanic, food insecure, separated/divorced/widowed, have lower educational attainment, household income below the poverty threshold, fewer social contacts, more depressive symptoms and live in food deserts (for more detail see Supplementary Material, Selection of Analytic Sample and Missing Data Analysis).

### Measures

#### Food and nutrient intake

For this study, seventeen food groups and nutrients associated with health in previous studies^(1–7,12)^ were examined: dairy products, fruits, vegetables, legumes, whole grains, protein, seafood, nuts and seeds, red and processed meat, dietary fibre, Ca, linoleic acid (*n*-6), linolenic acid (*n*-3), saturated fat, added sugar, Na and trans fat.

The HRS HCNS collected information about food consumption over the past 12 months using the Harvard Food Frequency Questionnaire originally developed by Willett and colleagues^(24,25)^, and the HRS team at the University of Michigan calculated average daily servings for each food item using nutrient tables provided by the Harvard School of Public Health (see Supplementary Material Calculation of the Average Daily Servings)^(24,26)^. For example, 1 serving/week is equivalent to 0·14 servings/d (1/7), and 5–6 servings/week is equivalent to 0·8 servings/d (5·5/7). The daily servings of food items were summed to create daily intake of dairy products, fruits, vegetables and protein and weekly intake of vegetable and protein foods subgroups (i.e. legumes, seafood, nuts and seeds, and red and processed meat). Raw data were used for whole grains and some nutrients (i.e. dietary fibre, Ca, linoleic acid, linolenic acid and Na). To assess intake of saturated fat, added sugar and trans-fat, the percentage of total daily energies from saturated fat, trans-fat and added sugar was calculated.

We also calculated the percentage of respondents who met dietary guidelines and nutritional goals for the seventeen food groups and nutrients based on respondents’ sex and age^(27)^. The optimal levels of intake for red and processed meat (<18 ounces/week) and trans fatty acids (<1 % of daily energies) are based on recommendations of the American Institute for Cancer Research^(28)^ and the American Heart Association^(29)^ as *the Dietary Guidelines for Americans* ^(27)^ does not provide this information. Detailed information on dietary assessment for the seventeen food and nutrients is available in the Supplementary Material: Dietary Assessment.

#### Diet quality

Overall diet quality was assessed using the Healthy Eating Index (HEI) 2015. While it was first developed in 1995 to assess overall dietary quality, the HEI has been updated several times based on US dietary guidelines and used in numerous nutrition studies^(30,31)^. The HEI-2015 contains thirteen components – total fruits, whole fruits, total vegetables, greens and beans, whole grains, dairy, total protein foods, seafood and plant proteins, fatty acids, refined grains, Na, added sugars and saturated fats – that sum to a total maximum score of 100 points. The thirteen components of the HEI-2015 are of two types: adequacy components and moderation components. Respondents get a high HEI-2015 score when they consume greater amounts of food and nutrients in the adequacy components (i.e. total fruits, whole fruits, total vegetables, greens and beans, whole grains, dairy, total protein foods, seafood and plant proteins, and fatty acids) and smaller amounts of food and nutrients in moderation components (i.e. refined grains, Na, added sugars and saturated fats). For this study, the HEI-2015 score was calculated using the simple HEI scoring algorithm method (see Supplementary Material Simple HEI Scoring Algorithm Method)^(32)^. An HEI score below 51 indicates a poor quality diet, scores between 51 and 80 reflect a diet that needs improvement and scores above 81 are considered a good quality diet^(31)^.

#### Sociodemographic, psychosocial, environmental and geographic factors

Potential protective and risk factors that have been related to diet quality include sociodemographic, psychosocial, environmental and geographic factors^(33,34)^. Older age, being female, being Hispanic and having higher socio-economic status have been associated with good quality diet^(15,35)^. Therefore, we included in our analysis age, sex, race/ethnicity (non-Hispanic White, non-Hispanic Black, Hispanic), education (less than high school (HS), HS diploma, college or above), poverty status and food insecurity. Poverty status was defined as having household income below the poverty threshold. Food insecurity status was assessed based on the short form of the U.S. Household Food Security Survey Module^(36)^, which includes six questions capturing self-perceived nutritional inadequacy, household food depletion, disrupted eating patterns and a repetitive pattern of reduced food intake^(37)^. Respondents were asked if the following two statements were often, sometimes or never true: ‘the food that we bought just didn’t last and we didn’t have enough money to get more’ and ‘we couldn’t afford to eat balanced meals’ (0 = never true, 1 = often true or sometimes true). Respondents were also asked if anyone in the household ever cut meal size or skipped meals because there was not enough money for food (0 = No, 1 = Yes) and if yes, how often it happened (0 = No or Yes, only 1 or 2 months, 1 = Yes, Some months but not every month or almost every month). Last, respondents were asked if they ever ate less than they felt they should because there was not enough money for it (0 = No, 1 = Yes) and they were ever hungry but did not eat because there was not enough money for food (0 = No, 1 = Yes). Responses to the items were summed (range: 0–6); raw score 0–1 is categorised as food security, and raw score 2–6 is considered food insecurity (see Supplemental Material: Food Insecurity Measure)^(36)^.

Psychosocial factors may also have an impact on diet quality as social networks and relationships can be valuable resources and sources of social and emotional support in later life. For example, rides provided by family and friends were one of the most common transportation modes for grocery shopping^(38)^. On the other hand, living alone, having infrequent social contacts and depression were associated with less healthful dietary behaviours as they demotivate cooking and healthy eating^(22,23,39)^. Based on previous findings, marital status (married/partnered, separated/divorced/widowed, never married), social contacts, level of social support and depression were included as psychosocial indicators. Social contacts were assessed based on the frequency of in-person meetings, phone calls and mail/email contacts with respondents’ non-resident children, other family and friends (range: 0 = Less than once a year or never – 5 = Three or more times a week). An index of social supports is an average score of seven items (three positively worded items and four negatively worded items; negatively worded items were reverse-coded) reflecting perceived social support from their spouses, children, family and friends where a greater value indicates high levels of social support (range: 0 = Not at all – 3 = A lot). Depression was assessed using the shortened Center for Epidemiologic Studies Depression scale^(40)^, which includes eight items (two positively worded items and six negatively worded items) with response options 0 = No and 1 = Yes. Responses to the items were summed (range: 0–8; positively worded items were reverse-coded), endorsing three or more on the eight items were classified as depressed^(40)^. Detailed information on the social contacts, positive/negative social supports and depression measures is available in Supplementary Material: Psychosocial Resources Measures.

Older adults may be more influenced by environmental factors due to decreased physical functioning. For example, older adults with limited independence and mobility may choose unhealthy food over healthy food because it is easier to get when a neighbourhood has limited access to supermarkets and grocery stores. Therefore, we included living in a food desert as a risk factor for suboptimal quality diet. We used the USDA-derived definition of food deserts, which combines low income and low access to sources of healthful food: a low-income census tract with at least 500 people or 33 % of the population living more than 1 mile (urban areas) or more than 10 miles (rural areas) from the nearest supermarket, supercentre or large grocery store^(41)^. Geographic region (Northeast, Midwest, South, West) is also included as it influences cultures which may influence food access, choice and intake^(17,42–44)^.

### Analysis plan

We first present the recommended amount of food and nutrients for adults over age 51 based on the dietary guidelines^(27–29)^ along with the average reported consumption of foods and nutrients among the HRS HCNS respondents and the percentage of those who consumed at least the recommended minimum amount of dairy, fruits, vegetables, legumes, whole grain, protein foods, seafood, nuts and seeds, dietary fibre, Ca, linoleic acid and linolenic acid and no more than the RDA of red and processed meats, saturated fat, added sugar, Na and trans-fat. We then compared differences in diet quality by sociodemographic, psychosocial, environmental and geographic factors using *χ* ^2^ and employed ordinary least squares regression to identify risk and protective factors for suboptimal quality diets. Sample weights provided by the HRS were applied in all analyses to account for the complex survey design. Analyses were conducted using Stata se 15^(45)^.

## Results

### Food and nutrient intake

Table 1 presents dietary guidelines and nutritional goals for older adults^(27–29)^ along with the average consumption of food and nutrients among the HRS HCNS respondents, and the percentage of those who consumed the optimal amount. The recommended amount reflects the maximum value for red and processed meat, saturated fat, added sugar, Na and trans-fat (2015–2020 Dietary Guidelines recommended limit; tolerable upper intake level) and the minimum amount for all other food groups and nutrients (RDA; adequate intake^(27)^). Average consumption of most food and nutrients of the HRS HCNS respondents was below the RDA or adequate intake amount. For example, older men and women consumed 1·30 (sd = 1·01) and 1·33 (sd = 1·21) cup-equivalent of dairy and 1·29 (sd = 0·96) and 1·21 (sd = 1·12) ounce-equivalent of whole grains, which is less than a half of the recommended amount (3 cup-equivalents of dairy; 3–3·5 ounce-equivalents of whole grains). On the other hand, the average consumption was above the recommended upper limit for red and processed meats (upper limit = <18 ounce-equivalent of red and processed meat; men M = 21·86, sd = 15·67; women M = 18·41, sd = 16·42), saturated fats (upper limit = <10 % of total energies; men M = 11·26, sd = 2·37; women M = 11·35, sd = 2·66) and added sugar (upper limit = <10 % of total energies; men M = 11·46, sd = 6·10; women M = 11·15, sd = 6·58).

**Table 1:** Dietary guidelines and nutritional goals for older adults (51+), the average daily/weekly consumption of food and nutrients and the percentage of older adults who consumed the optimal amount of food and nutrients among a nationally representative sample of older Americans in the HRS HCNS (*n* 5614)

	Recommended amount*	Men (*n* 2307)			Recommended amount*	Women (*n* 3307)		
		M	sd	% Optimal		M	sd	% Optimal
Food groups								
Dairy, cup-equivalent	3	1·30	1·01	8·0	3	1·33	1·20	8·0
Fruits, cup-equivalent‡	2	1·21	0·98	18·0	1·5	1·30	1·29	30·2
Vegetables, cup-equivalent‡	2·5–3	1·59	0·98	13·2	2	1·79	1·26	34·0
Legumes‡	1·5–2	0·64	0·66	9·7	1	0·70	0·85	15·3
Grains, ounce-equivalent								
Whole grains	3–3·5	1·29	0·96	5·0	3	1·21	1·12	4·2
Protein, ounce-equivalent‡	5·5–6	6·44	3·52	50·0	5	6·04	4·04	53·7
Seafood	8–9	5·31	5·14	15·6	8	5·38	6·60	17·9
Nuts and seeds	5	6·94	8·07	43·5	4	6·69	8·94	45·5
Red and processed meat‡	<18 oz	21·86	15·67	52·2	<18 oz	18·41	16·42	63·2
Nutrients								
Dietary fibre, g‡	28	19·00	9·78	23·8	22·4	19·81	12·93	30·0
Ca, mg‡	1000–1200†	740·27	454·82	16·9	1200	780·67	679·72	12·5
Polyunsaturated fat								
Linoleic acid (*n*-6), g‡	14	12·27	6·98	31·0	11	11·83	8·82	42·3
Linolenic acid (*n*-3), g‡	1·60	1·38	0·82	27·6	1·10	1·41	1·11	53·5
Saturated fat, % kcal	<10 %	11·26	2·37	30·0	<10 %	11·35	2·66	30·1
Added sugar, % kcal	<10 %	11·46	6·10	49·2	<10 %	11·15	6·58	51·0
Na, mg‡	<2300	2295·66	1087·60	57·1	<2300	2127·27	1341·21	67·3
Trans-fat, % kcal	<1 %	0·71	0·21	91·8	<1 %	0·66	0·22	94·0

* Recommended amount of dietary intake for men aged 51–60 years at 2200 energy level, men over age 61 years at 2000 energy level and women over age 51 years at 1600 energy level^(22–24)^. Food amounts shown in cup-equivalents for dairy, fruit, and vegetable groups and ounce-equivalents for grains and protein groups. Vegetable and protein foods subgroup amounts (i.e. legumes, seafood, nuts and seeds, and red and processed meat) are recommended weekly intake amounts, and all other food and nutrient amounts are recommended daily intake amounts. The recommended amount for red and processed meat, saturated fat, added sugar, Na and trans-fat is maximum (DGA; UL), and the amount for all other food groups and nutrients is minimum (RDA; AL).

† For men over age 71.

‡ Significant differences in the percentage consumed the optimal amount at *P* < 0·05.

Overall, most respondents did not meet the dietary guidelines and nutritional goals for most food and nutrients. Intake of dairy products and whole grains was very low; <10 % of respondents met the dietary recommendation for these food groups (men 5·0 %; women 4·2 %). In addition, <30 % of the respondents met dietary guidelines for fruits (only among men 18·0 %), vegetables (only among men 13·2 %), legumes (men 9·7; women 15·3 %), seafood (men 15·6 %; women 17·9 %), dietary fibre (only among men 23·8 %), Ca (men 16·9 %; women 12·5 %) and linolenic acid (only among men 27·6 %).

The suboptimal diet was more frequently observed among men. A significantly smaller percentage of male respondents consumed the optimal amount of fruits (men 18·0 %; women 30·2 %; *P* < 0·001), vegetables (men 13·2 %; women 34·0 %; *P* < 0·001), legumes (men 9·7 %; women 15·3 %; *P* < 0·001), protein (men 50·0 %; women 53·7 %; *P* < 0·05), red and processed meat (men 52·2 %; women 63·2 %; *P* < 0·001), dietary fibre (men 23·8 %; women 30·0 %; *P* < 0·001), Ca (men 16·9 %; women 12·5 %; *P* < 0·001), linoleic acid (men 31·0 %; women 42·3 %; *P* < 0·001), linolenic acid (men 27·6 %; women 53·5 %; *P* < 0·001), Na (men 57·1 %; women 67·3 %; *P* < 0·001) and trans-fat (men 91·8 %; women 94·0 %; *P* < 0·01).

### Sociodemographic, psychosocial, environmental and geographic factors and diet quality

Table 2 shows differences in overall diet quality by sociodemographic, psychosocial and environmental factors. The diet quality is based on the HEI score, a multicomponent score that is used to assess overall quality rather than separate indicators of diet. The mean HEI-2015 score was 66·93 (sd = 11·34). The majority of the respondents had suboptimal quality diets, 8·4 % with a poor quality diet (HEI-2015 score below 51) and 80·7 % with a diet that needs improvement (HEI-2015 score between 51 and 80); only 11·0 % of the respondents had a good quality diet (HEI-2015 score above 81). Poor quality diet is more common for males (male 10·9 %; female 7·1 %; *P* < 0·001), non-Hispanic Whites and Blacks (non-Hispanic White 9·2 %; non-Hispanic Black 8·2 %; Hispanic 4·4 %; *P* < 0·01), the food insecure (food insecure 13·7 %; food secure 7·8 %; *P* < 0·001), with lower educational attainment (less than HS 12·4 %; HS 12·0 %; HS+ 5·9 %; *P* < 0·001) and with household income below the poverty threshold (in poverty 14·9 %; not in poverty 8·2 %). Those who reported poor quality diet were also less likely to be married (never married 12·8 %; separated/divorced/widowed 10·1 %; married/partnered 7·7 %; *P* <0·05) and reported less frequent social contacts (infrequent social contacts 11·0 %; frequent social contacts 6·5 %; *P* < 0·001), low levels of social support (low levels of social support 9·5 %; high levels of social support 8·0 %; *P* < 0·05) and more depression (depression 12·5 %; no depression 8·0 %; *P* < 0·001). In addition, respondents who lived in food deserts were more likely to have a poor quality diet than those who did not (food desert 10·1 %; not food desert 8·6 %; *P* < 0·01), as were people who lived in the Midwest and South (Midwest 10·6 %; South 9·7 %; Northeast 7·3 %; West 5·7 %; *P* < 0·001).

**Table 2:** Sociodemographic, psychosocial, environmental and geographic factors and diet quality in a nationally representative sample of older Americans: HRS HCNS (*n* 5614)

	Poor diet (*n* 468) (%)	Needs improvement (*n* 4525) (%)	Good diet (*n* 615) (%)		Poor diet (*n* 468) (%)	Needs improvement (*n* 4525) (%)	Good diet (*n* 615) (%)
Sociodemographic factors				Psychosocial factors			
Age				Marital status*			
Age 54–63	9·6	79·6	10·8	Married	7·7	82·1	10·2
Age 64–73	8·5	81·6	9·9	Separated/divorced/widowed	10·1	79·4	10·6
Age 74–83	7·0	81·6	11·4	Never married	12·8	74·1	13·1
Age 84+	8·9	82·0	9·1	Social contacts*,†			
Sex*				Infrequent social contacts	11·0	80·6	8·4
Male	10·9	81·3	7·8	Frequent social contacts	6·5	80·9	12·6
Female	7·1	80·3	12·6	Social support*,‡			
Race*				Low levels of social support	9·5	81·3	9·2
White	9·2	80·4	10·4	High levels of social support	8·0	80·3	11·7
Black	8·2	83·0	8·8	Depression*			
Hispanic	4·4	82·2	13·4	No depression	8·0	80·6	11·5
Education*				Depression	12·5	81·8	5·7
Less than HS	12·4	81·2	6·4	Environmental factor			
HS	12·0	79·9	8·1	Food desert*,§			
HS+	5·9	81·2	12·8	No	8·6	80·4	11·0
Poverty status*				Yes	10·1	83·0	7·0
Not in poverty	8·2	81·0	10·8	Geographic factor			
In poverty	14·9	79·7	6·4	Region*			
Food insecurity*				Northeast	7·3	80·3	12·4
Food secure	7·8	81·0	11·1	Midwest	10·6	81·8	7·6
Food insecure	13·7	79·4	6·9	South	9·7	81·3	9·0
				West	5·7	78·8	15·5

An HEI score below 51 indicates a poor quality diet, scores between 51 and 80 reflect a diet that needs improvement and scores above 81 are considered a good quality diet^(31)^. The average HEI score for the sample was 66·93 (sd = 11·34).

* Significant differences between groups at *P* < 0·05.

† The original variable assesses the frequency of in-person meeting and contacts via phone, write and email with respondents’ non-resident children, other family and friends (0 = Less than once a year or never 5 = Three or more times a week). For the bivariate analysis, the variable was dichotomised using 50th percentile as a cut-point.

‡ The original variable assesses perceived social support from respondents’ spouses, children, family and friends (0 = Low levels of social support–3 = High levels of social support). For the bivariate analysis, the variable was dichotomised using 50th percentile as a cut-point.

§ Food desert: A low-income tract with at least 500 people or 33 % of the population living more than 1 mile (urban areas) or more than 10 miles (rural areas) from the nearest supermarket, supercentre or large grocery store.

We estimated an ordinary least squares regression model to identify risk and protective factors for good quality diet when all variables are considered at once. Table 3 presents the results of the ordinary least squares regression model for the association between sociodemographic, psychosocial, environmental and geographic factors and diet quality. Older age (b = 0·06, 95 % CI 0·02, 0·10), being female (b = 2·48, 95 % CI 1·75, 3·21), being Black (b = 1·89, 95 % CI 0·78, 2·99), being Hispanic (b = 5·08, 95 % CI 3·76, 6·41), higher educational level (more than HS) (b = 3·86, 95 % CI 2·73, 5·00), frequent social contacts (b = 1·10, 95 % CI 0·63, 1·58), high levels of social support (b = 1·18, 95 % CI 0·20, 2·16) and being Western residents (b = 1·82, 95 % CI 0·65, 2·99) were positively associated with HEI scores. On the other hand, respondents who were food insecure (b = –1·88, 95 % CI –2·96, –0·80), were separated/divorced/widowed (b = –0·81, 95 % CI –1·63, 0·02), had depression (b = –2·24, 95 % CI –3·23, –1·26) and lived in a food desert (b = −1·17, 95 % CI –2·15, –0·19), Midwest (b = –1·56, 95 % CI –2·68, –0·45) and South (b = –1·25, 95 % CI –2·30, –0·19) were more likely to have lower HEI scores, which indicate poor or suboptimal quality diet. Poverty status was not statistically significantly associated with diet quality once the other variables were controlled.

**Table 3:** Coefficients from ordinary least squares regression predicting diet quality (Healthy Eating Index 2015 score) (*n* 5614)

	Coefficient	95 % CI	
Sociodemographic factors			
Age (range: 53–99)	0·06 ***	0·02	0·10
Sex (Ref: Male)			
Female	2·48 ****	1·75	3·21
Race (Ref: White)			
Black	1·89 ****	0·78	2·99
Hispanic	5·08 ****	3·76	6·41
Education (Ref: Less than HS)			
HS	0·70	−0·44	1·84
HS+	3·86 ****	2·73	5·00
Poverty status (Ref: Not in poverty)			
In poverty	−1·01	−2·42	0·41
Food insecurity (Ref: Food secure)			
Food insecure	−1·88 ****	−2·96	−0·80
Psychosocial factors			
Marital status (Ref: Married/Partnered)			
Separated/divorced/widowed	−0·81 *	−1·63	0.02
Never married	−1·14	−2·88	0·61
Social contacts (range:0–5)†	1·10 ****	0·63	1·58
Social support (range: 0–3)‡	1·18 **	0·20	2·16
Depression	−2·24 ****	−3·23	−1·26
Environmental factor			
Food desert (Ref: No)||			
Yes	−1·17 **	−2·15	−0·19
Geographic factor			
Region (Ref: Northeast)			
Midwest	−1·56 ***	−2·68	−0·45
South	−1·25 **	−2·30	−0·19
West	1·82 ***	0·65	2·99
Constant	54·60 ****	50·86	58·34
*R* ^2^	0·10		

* *P* < 0·10, **P < 0·05, ***P < 0·01, ****P < 0·001.

† In-person meeting and contacts via phone, write and email with respondents’ non-resident children, other family and friends were measured as: 0 = Less than once a year or never; 1 = Once or twice a year; 2 = Every few months; 3 = Once or twice a month; 4 = Once or twice a week; 5 = Three or more times a week.

‡ Perceived social support from respondents’ spouses, children, family and friends was measured as: 0 = A lot; 1 = Some; 2 = A little; 3 = Not at all.

|| Food desert: A low-income tract with at least 500 people or 33 % of the population living more than 1 mile (urban areas) or more than 10 miles (rural areas) from the nearest supermarket, supercentre or large grocery store.

## Discussion

The purpose of this study was to understand dietary intake of food and nutrients and assess dietary quality among older Americans based on a nationally representative sample of community-dwelling older adults. Findings of this study suggest that dietary patterns of older Americans are far from optimal. The intake of core food groups, dietary fibre, Ca and polyunsaturated fat (among men) was lower than the minimum recommended amount and the intake of red and processed meats, saturated fats and added sugar was above the maximum recommended upper limit. In addition, the majority of older adults did not meet dietary recommendations for most food and nutrients. However, over 90 % of the respondents consumed less than the recommended maximum intake of trans-fat. The use of trans-fat is regulated by the Food and Drug Administration. Since it was first banned in New York City in 2006, the amount of trans-fats in food products as well as the average intake of trans-fats has decreased significantly^(46,47)^. This example suggests that state- or federal-level food regulations may be an effective way to decrease suboptimal dietary intakes^(46,48)^.

Older women had diets somewhat better matched to dietary guidelines and nutritional goals, compared with older men, which is consistent with previous studies indicating that women eat healthier than men^(15,17)^. Previous studies have suggested men’s limited nutrition-related knowledge and cooking skills as primary reasons for choosing convenient and unhealthy food, which is often not fresh and less nutritious^(15,17)^. Therefore, provision of food and nutrition education that can increase food and nutrition-related knowledge and enhance their cooking skills would improve diet quality of older men.

Respondents with low socio-economic status were more likely to have lower HEI-2015 scores or poor/suboptimal quality diet. The negative association between low educational attainment and diet quality has been consistently reported^(15,49)^. A link between educational level and nutrition knowledge and dietary behaviour may be a primary reason for the association^(15,50)^. The negative associations between poverty and food insecurity and diet quality have also been frequently reported^(15,16,18,51)^, although the association was weak or not significant in some studies^(15,35)^. In our sample, food insecurity was significantly associated with lower HEI-2015 scores, while poverty status was not significantly associated with HEI-2015 scores reflecting diet quality. This may be because food insecurity reflects financial hardship that influences food purchase and intake and eating patterns, while poverty captures broader financial difficulties. Previous studies have reported that the Supplemental Nutrition Assistance Program can reduce food insecurity^(52–54)^. Therefore, expanding the Supplemental Nutrition Assistance Program and encouraging enrolment may help promote diet quality and health of food-insecure older adults.

Despite their low socio-economic status, Hispanics and Blacks had higher HEI-2015 scores, compared with Whites. The healthier diet among Hispanics, including higher intake of fibre and polyunsaturated fat and lower intake of refined grains, saturated fat and added sugars, has been reported in previous studies^(55,56)^. This may be because traditional Mexican food contains more fruit, vegetables, legumes and whole grains^(56,57)^. In addition, multigenerational living which is prevalent among older Hispanics may promote healthy eating^(58)^. There is little consensus on dietary differences between Blacks and Whites, although more unhealthy dietary patterns have been observed among Blacks. Previous studies have reported poorer diet quality and lower intake of dairy, vegetables, fibre and Ca among Blacks, compared with Whites^(59,60)^. However, healthier dietary patterns, including greater intake of polyunsaturated fat and lower intake of saturated fat and Na, were also observed among Blacks^(59,60)^.

Psychosocial factors, including social contacts, social support and depression, are also associated with diet quality. Respondents with more psychosocial resources were more likely to have higher HEI-2015 scores or good quality diet. Previous studies have reported that while age-related physical changes (e.g. loss of physical functioning and mobility) prevent older adults’ access to healthy foods and intake of well-balanced, nutritious diets^(17)^, social relationships and networks provide instrumental support, such as provision of transportation and help with grocery shopping and meal preparation^(38,61)^. Also, frequent social contacts and gatherings prevent older adults from eating alone, which motivates cooking and eating, whereas social isolation and depression were associated with unhealthy dietary intake and nutritional risk^(18,39,62)^. Increasing opportunities to build social relationships and networks, by providing more social activities and events for example, may improve diet quality of older adults.

Residents of a food desert were more likely to have lower HEI-2015 scores or poor/suboptimal quality diet. The negative impact of living in a food desert or a neighbourhood with limited access to healthy food on food insufficiency and unhealthy eating behaviours (e.g. meal skipping) has been reported in previous studies^(63)^. Respondents who lived in the Midwest and South were also more likely to have lower HEI-2015 scores or poor/suboptimal quality diet, compared with those who lived in the Northeast. The unhealthy dietary intake and poor quality diet of Midwest and South, including low consumption of fruits, vegetables, whole grains and dietary fibre and high intake of energy-dense foods, fat and added sugar, have been reported in previous studies^(42–44)^. Various factors that are related to food access, such as high rates of poverty, low education and racial/ethnic background of the residents, as well as lower access to certain food groups and higher prices for the same products due to geographic location and limited store availability may have contributed to the regional differences^(17,35,42,64)^. Therefore, increasing the number of healthy food outlets (grocery stores and farmers’ markets) that provide affordable food would improve access to fresh, healthy food and promote diet quality of older residents^(63,65)^.

### Limitations

Although this study has important implications, it has several limitations worth noting. First, dietary intake was assessed based on the average intake of food and nutrients during the past 12 months. This may have introduced recall bias into this study, and the results may not reflect actual dietary intake. Also, the Dietary Guidelines for Americans^(27)^ and the HRS HCNS used different measurement units. To minimise potential bias, we converted the measurement units carefully based on the guidelines and compared the results with the National Health and Nutrition Examination Survey, which has been collecting information on diet and nutritional status through 24-h recall method using the same measurement units as the Dietary Guideline. The average intake of food and nutrients between the HRS respondents and the National Health and Nutrition Examination Survey was similar. Lastly, this study is based on 2012 and 2013 data. Although diet quality or dietary patterns do not generally change^(66)^, further research should be conducted to confirm findings of this study.

## Conclusion

Healthful eating is essential for the prevention of chronic diseases and promotion of health.

This study highlights the need for improving diet among older Americans. The majority of older adults did not meet dietary recommendations for core food groups and nutrients. Especially, respondents with lower socio-economic status, fewer psychosocial resources and limited access to grocery markets and stores had less healthful dietary patterns and poor quality diet. Dietary policies and interventions that focus on promoting food and nutrition literacy through education and marketing increase access to healthy food (e.g. financial assistance, healthy food outlets, transportation options and delivered meals), while those discouraging unhealthful eating (e.g. food regulations and taxes) may improve older adults’ diet quality. In addition, more opportunities to increase social relationships and networks may provide social, emotional and instrumental supports for healthy eating.

Given their higher risk for severe illness and social isolation, the COVID-19 pandemic may have significant impact on older adults and their lifestyle behaviours, including diet^(67)^. Therefore, supportive policies and services, such as designated shopping hours and delivery services for older adults, may increase access to healthy foods and help older adults maintain healthy eating.

## Supplementary material

For supplementary material accompanying this paper visit https://doi.org/10.1017/S1368980021000586. click here to view supplementary material
