# Supplementary material for: Food and nutrient intake and diet quality among older Americans
Source: Public Health Nutr. 2021 Feb 9;24(7):1638–47. doi: 10.1017/S1368980021000586 (PMC8094430; doi:10.1017/S1368980021000586)
Supplement: Supplementary file 1 [file S1368980021000586sup001.docx]

**Supplementary Material: Selection of Analytic Sample and Missing Data Analysis**

This study used data from the 2013 Health Care and Nutrition Survey (HCNS), a sub-study of the Health and Retirement Study (HRS), that includes a random subsample of the HRS respondents and their spouse/partners (N=8,035). In 2013 the sample was age 54 and older. Since the 2013 HCNS includes a subsample of the HRS respondents and their spouse/partner regardless of their age, we excluded those who were below age 54 when interviewed to only include age-eligible respondents. Among 7,383 age-eligible respondents, respondents who could not be linked with psychosocial resources data and food environment data (N=260) and those with missing values on key variables and covariates (N=1,509) were excluded from the sample. The final analytic sample includes 5,614 respondents.

Figure A. Sample Inclusion Criteria

All Respondents in the Health and Retirement Health Care and Nutrition Survey (N=8,035)

Respondents aged 54+

(N=7,383)

Respondents with psychosocial resources data and food environment data

(N=7,123)

1,509 respondents have missing values for at least one of the included variables.

Analytic sample

(N=5,614)

Table A presents participants characteristics for respondents with complete data and respondents with missing data.

Table A. Participants characteristics

|  | Complete Data | | Missing Data |  | Complete Data | | Missing Data |
| --- | --- | --- | --- | --- | --- | --- | --- |
|  | % | | % |  | % | | % |
| ***Sociodemographic Factors*** | | | | ***Psychosocial Factors*** |  | |  |
| Age ^a^ |  | |  | Marital Status ^a^ |  | |  |
| Age 54-63 | 48.5 | 50.6 | | Married | 63.4 | 58.1 | |
| Age 64-73 | 29.9 | 24.8 | | Separated/Divorced/Widowed | 30.4 | 36.4 | |
| Age 74-83 | 15.2 | 15.6 | | Never married | 6.1 | 5.5 | |
| Age 84+ | 6.4 | 7.1 | | Social contacts ^a^ |  |  | |
| Sex ^a^ |  |  | | Infrequent social contacts | 49.2 | 59.0 | |
| Male | 45.0 | 49.2 | | Frequent social contacts | 50.8 | 41.0 | |
| Female | 55.0 | 50.8 | | Social support |  |  | |
| Race ^a^ |  |  | | Low levels of social support | 50.8 | 48.7 | |
| White | 84.6 | 70.5 | | High levels of social support | 49.2 | 51.3 | |
| Black | 8.7 | 15.5 | | Depression ^a^ |  |  | |
| Hispanic | 6.8 | 14.0 | | No depression | 82.6 | 74.1 | |
| Education ^a^ |  |  | | Depression | 17.4 | 25.9 | |
| Less than HS | 12.8 | 22.7 | | ***Environmental Factor*** |  |  | |
| HS | 32.9 | 28.9 | | Food desert |  |  | |
| HS+ | 54.3 | 48.4 | | No | 55.0 | 56.7 | |
| Poverty status ^a^ |  |  | | Yes | 45.0 | 43.3 | |
| Not in poverty | 91.7 | 86.8 | | ***Geographic Factor*** |  |  | |
| In poverty | 8.3 | 13.3 | | Region ^a^ |  |  | |
| Food insecurity ^a^ |  |  | | Northeast | 16.2 | 14.4 | |
| Food secure | 84.0 | 75.9 | | Midwest | 27.0 | 22.0 | |
| Food insecure | 16.0 | 21.2 | | South | 36.4 | 43.4 | |
|  |  |  | | West | 20.4 | 19.1 | |

^a^ Significant diet quality differences at *p*<.05.

We ran a weighted logistic regression to examine whether the respondents who were excluded from this study differed from the analytic sample. Odds ratios and significance are reported in Table B.

Table B. Weighted Logistic Regression Model Predicting Being Excluded from the Analytic Sample

|  | Odds Ratio (SE) | |
| --- | --- | --- |
| Age | 1.00 (0.01) |  |
| Sex (Ref: Male) |  |  |
| Female | 0.84 (0.05) | ** |
| Race (Ref: White) |  |  |
| Black | 2.04 (0.14) | *** |
| Hispanic | 2.45 (0.19) | *** |
| Education (Ref: Less than HS) |  |  |
| HS | 0.54 (0.04) | *** |
| HS+ | 0.51 (0.04) | *** |
| Poverty status (Ref: Not in poverty) |  |  |
| In poverty | 1.92 (0.16) | *** |
| Food insecurity (Ref: Food secure) |  |  |
| Food insecure | 1.66 (0.12) | *** |
| Marital Status (Ref: Married/Partnered) |  |  |
| Separated/Divorced/Widowed | 1.34 (0.08) | *** |
| Never married | 1.07 (0.15) |  |
| Social contacts (range:0-5) | 0.82 (0.04) | *** |
| Social support (range: 0-3) | 1.00 (0.11) |  |
| Depression | 1.12 (0.02) | *** |
| Food desert (Ref: No) ^c^ |  |  |
| Yes | 0.89 (0.05) | * |
| Region (Ref: Northeast) |  |  |
| Midwest | 0.76 (0.07) | ** |
| South | 1.21 (0.10) | * |
| West | 1.04 (1.00) |  |

**p*<.05, ***p*<.01, ****p*<.001.

Respondents with missing data were more likely to be male, Black, Hispanic, food insecure, separated/divorced/widowed, have lower educational attainment, household income below the poverty threshold, fewer social contacts, more depressive symptoms, and live in food deserts.

**Supplementary Material: Calculation of the Average Daily Servings ^(19)^**

The HRS team at the University of Michigan calculated average daily servings for each food item. For the calculation, the food frequency data were converted from a categorical response to a numeric value reflecting servings per day using Harvard University’s food serving conversion guides. For example, 1 serving per week is equivalent to 0.14 servings per day (1/7), and 5-6 servings per week is equivalent to 0.8 servings per day (5.5 / 7). The source files used for deriving the data were obtained from the Harvard University School of Public Health’s download site: <https://regepi.bwh.harvard.edu/health/nutrition.html>

**Supplementary Material: Simple HEI Scoring Algorithm Method ^(27)^**

The steps of the HEI scoring algorithm method are:

1. **Deriving Sums**. For each HEI component, dietary constituents are summed together (e.g., Greens and Beans is created from the sum of dark green vegetables and legumes (beans and peas)).
2. **Constructing Ratios**. The appropriate ratios are constructed for each individual. Usually these are the ratios of the dietary constituents to 1000 kcal of energy, with the exception of fatty acids, which use the ratio of the sum of monounsaturated and polyunsaturated fatty acids to saturated fatty acids. (Also, note two components are expressed on a percent of calories basis. Therefore, grams of saturated fat should be multiplied by 9 to convert g to kcal, and added sugars should be multiplied by 16 to convert teaspoons to kcal, prior to dividing by total energy.)
3. **Scoring**. The ratios are scored according to the HEI scoring standards for each component. The component scores are summed to calculate the total score.

**Supplementary Material: Dietary Assessment ^(22)^**

The 2015-2020 *Dietary Guidelines for Americans* ^(22)^ was used to make decisions regarding the inclusion of food items within the main food groups and the subgroups. Foods in each group and subgroup, listed in *the Dietary Guidelines for Americans*, are as follow:

- *Dairy* included all milk (including soymilk), yogurt, frozen yogurt, dairy desserts, and cheeses. 1 cup milk and yogurt, 1.5 ounces of natural cheese, or 2 ounces of processed cheese were considered as 1 cup-equivalent dairy. Cream and cream cheese were excluded due to their low calcium content.
- *Fruits* included all fresh, frozen, canned, and dried fruits and fruit juices. 1 cup of raw fruit, 1 cup of fruit juice, or 0.5 cup of dried fruit were considered as 1 cup-equivalent fruit.
- *Vegetables* included all fresh, frozen, canned vegetables, cooked or raw. 1 cup of raw or cooked vegetable, 1 cup of vegetable juice, 2 cups of leafy salad greens, or 0.5 cup of dried vegetable were considered as 1 cup-equivalent vegetable.
- *Legumes*, a sub-group of the vegetable group, included all cooked from dry or canned beans and peas and soy products (e.g., tofu). Legumes and soy products can be considered either the vegetable group or the protein group, but should be counted in one group only ^(22)^. In this study, they were counted in the vegetable group.
- *Seafood*, a sub-group of the protein group, included all seafood, including canned fish. 1 ounce of seafood was considered as 1 ounce-equivalent seafood.
- *Red and processed meat*, a sub-group of the protein group, included all red meats (e.g., beef, pork, and lamb) and processed meats (e.g., bacon, sausages, and salami). 1 ounce of red and processed meat was considered as 1 ounce-equivalent red and processed meat.
- *Nuts and seeds*, a sub-group of the protein group, included all nuts, seeds, and related products (e.g., peanut butter). 0.5 ounce of nuts or seeds or 1 tablespoon of peanut butter were considered as 1 ounce equivalent nuts and seeds.

The number of servings of food items were then summed to create daily intake of dairy products, fruits, vegetables, and protein and weekly intake of vegetable and protein foods subgroups (i.e., legumes, seafood, nuts, seeds, and soy products, and red and processed meat).

The raw data were used for whole grains, nutrients, and minerals.

The percentage of total daily calories from saturated fat, trans fat, and added sugar were calculated as:

$$The percentage of total daily calories from saturated/trans fat=\frac{saturated/trans fat\times9kcal}{total daily calories}\times100$$

$$The percentage of total daily calories from added sugar=\frac{added sugar\times4kcal}{total daily calories}\times100$$

**Supplementary Material: Food Insecurity Measure**

Food insecurity status was assessed based on the short form of the U.S. Household Food Security Survey Module ^(28)^.

| **Questions** | **Response Options & Scores** |
| --- | --- |
| Q1. The food that we bought just didn't last and we didn't have enough money to get more | Often (1)  Sometimes (1)  Never (0) |
| Q2. We couldn’t afford to eat balanced meals. | Often (1)  Sometimes (1)  Never (0) |
| Q3. In the last twelve months, did you or other adults in your household ever cut the size of your meals or skip meals because there wasn't enough money for food? | Yes (1)  No (0) |
| Q4-Q5. In the last twelve months, did you ever eat less than you felt you should because there wasn't enough money for food? If yes, how often did it happen? | Yes, almost every month (2)  Yes, some months but not every month (2)  Yes, only 1 or 2 months (1)  No (0) |
| Q6. In the last twelve months, were you ever hungry but didn't eat because there wasn't enough money for food? | Yes (1)  No (0) |

Responses to the six questions in the module were summed (range: 0-6). Raw score 0-1 categorized as food security and raw score 2-6 is considered food insecurity ^(28)^.

**Supplementary Material: Psychosocial Resources Measures**

**Social Contacts**

Nine questions assess the extent to which respondents are in contact with their social networks (i.e., children, other family, and friends).

| **Questions** |
| --- |
| On average, how often do you do each of the following with your children, not counting any who live with you?  Q1. Meet up (include both arranged and chance meetings)  Q2. Speak on the phone  Q3. Write or email |
| On average, how often do you do each of the following with other family members, not counting any who live with you?  Q4. Meet up (include both arranged and chance meetings)  Q5. Speak on the phone  Q6. Write or email |
| On average, how often do you do each of the following with any of your friends, not counting any who live with you?  Q7. Meet up (include both arranged and chance meetings)  Q8. Speak on the phone  Q9. Write or email |

Response options include: 0 = Less than once a year or never, 1 = Once or twice a year, 2 = Every few months, 3 = Once or twice a month, 4 = Once or twice a week, and 5 = Three or more times a week. Responses were averaged to create a measure of overall contact with the social network (range: 0-5).

**Perceived Social Support**

Four sets of seven items (three positively worded items and four negatively worded items) examine the perceived support that respondents receive from their spouses, children, family, and friends.

| **Questions** |
| --- |
| Q1. How much do they really understand the way you feel about things?  Q2. How much can you rely on them if you have a serious problem?  Q3. How much can you open up to them if you need to talk about your worries?  Q4. How often do they make too many demands on you?  Q5. How much do they criticize you?  Q6. How much do they let you down when you are counting on them?  Q7. How much do they get on your nerves? |

Response options include 0 = Not at all, 1 = A little, 2 = Some, and 3 = A lot. Q4, Q5, Q6, and Q7 were reverse-coded. Responses were averaged to create an index of social support where a greater score indicates high levels of social support (range: 0-3).

**Depression**

Depression was assessed using the shortened Center for Epidemiologic Studies Depression scale ^(33)^. Respondents were asked to answer if the following eight statements were true.

| **Questions** |
| --- |
| Much of the time during the past week…  Q1. You felt depressed.  Q2. You felt that everything you did was an effort.  Q3. Your sleep was restless.  Q4. You were happy.  Q5. You felt lonely.  Q6. You enjoyed life.  Q7. You felt sad.  Q8. You could not get going. |

Response options include: 0 = No and 1 = Yes. Q4 and Q6 were reverse-coded. Responses to the items were summed (range: 0-8). Three or more on the eight items were classified as depression ^(34)^.
